# Determinants of wife-beating acceptance among reproductive age women in Ethiopia: a multilevel analysis of 2016 Ethiopian demographic and health survey

**DOI:** 10.1186/s12905-021-01484-1

**Published:** 2021-09-27

**Authors:** Mastewal Arefaynie, Gedamnesh Bitew, Erkihun Tadesse Amsalu, Bereket Kefale, Amare Muche, Zinabu Fentaw, Reta Dewau, Mequannent Sharew Melaku, Melaku Yalew, Bezawit Adane, Metadel Adane, Muluken Genetu Chanie, Wolde Melese Ayele, Yitayish Damtie

**Affiliations:** 1 Department of Reproductive and Family Health, School of Public Health, College of Medicine and Health Sciences, Wollo University, PO Box: 1145, Dessie, Ethiopia; 2 Department of Epidemiology and Biostatistics, School of Public Health, College of Medicine and Health Sciences, Wollo University, Dessie, Ethiopia; 3 Department of Health Informatics, Institute of Public Health, College of Medicine and Health Sciences, University of Gondar, Gondar, Ethiopia; 4 Department of Environmental Health, College of Medicine and Health Sciences, Wollo University, Dessie, Ethiopia; 5 Department of Health Systems and Policy, College of Medicine and Health Sciences, Wollo University, Dessie, Ethiopia

**Keywords:** Acceptance, Wife beating, Women, Multi-level analysis, EDHS 2016

## Abstract

**Background:**

There is limited national representative evidence on determinants of women’s acceptance of wife-beating especially; community level factors are not investigated in Ethiopia. Thus, this study aimed to assess individual and community-level factors associated with acceptance of wife beating among reproductive age women in Ethiopia.

**Methods:**

Secondary data analysis was done on 2016 Ethiopian Demographic and Health Survey data. A total of 15,683 weighted reproductive age group women were included in the analysis. Multi-level mixed-effect logistic regression analysis was done by Stata version 14.0 to identify individual and community-level factors. An adjusted odds ratio with a 95% confidence interval was used to show the strength and direction of the association. Statistical significance was declared at *p* value less than 0.05 at the final model.

**Result:**

Individual-level factors significantly associated with acceptance of wife-beating among women were; being Muslim follower [AOR = 1.3, 95% CI = (1.1, 1.5)], Being married [AOR = 1.3, 95% CI = (1.1, 1.6)], attending primary, secondary and higher education [AOR = 0.8, 95% CI = (0.7, 0.9)], [AOR = 0.4, 95% CI = (0.3, 0.5)], [AOR = 0.3, 95% CI (0.2, 0.4)] respectively. From community level factors, living in Somali [AOR = 0.2 95% CI = (0.1, 0.3)], Addis Ababa [AOR = 0.3, 95%CI = (0.2, 0.5)] and Dire Dawa [AOR = 0.5, 95% CI = (0.3, 0.7)] were 80%, 70% and 50% less likely accept wife-beating when compare to women who live in Tigray region, respectively. Live in high proportion of poor community [AOR = 1.2, 95% CI = (1.1, 1.3)], live in low proportion of television exposure communities [AOR = 1.4, 95% CI = (1.2, 2.2)] were significantly associated with acceptance of wife-beating among women in Ethiopia.

**Conclusion:**

Educational status, religion, marital status, region, community-level wealth, and community level of television exposure had a statistical association with women’s acceptance of wife-beating. Improving educational coverage, community-level of media exposure, community-level wealth status and providing community-friendly interventions are important to reduce the acceptance of wife-beating among women in Ethiopia.

## Background

Gender-based violence (GBV) is any harm or suffering that is perpetrated against a woman or girl, man or boy and that harms the physical, sexual or psychological health, development, or identity of the person [1–4]. GBV is a widespread human rights issue, which is critical in gender inequality and disproportionately affected women and girls globally [5–9]. Violence restricts the ability of women and girls to fully participate in and contribute to communities politically, economically, and socially. Women and young girls are disproportionally affected by physical and sexual violence [10–12]. Wife beating is common throughout the world [13–21]. The burden ranges from 15 to 79% [22]. Partner violence occurs in all countries and transcends social, economic, religious, and cultural groups. Worldwide, one of the most common forms of violence against women is abuse by their husbands or other intimate male partners [23].

Intimate partner violence is high when society accepts it [24–26]. Beliefs and norms seem to grant men control over female behavior, making violence acceptable for resolving conflicts [27]. Other studies have suggested that, in societies with a high prevalence of interpersonal violence, attitudes that encourage or tolerate violence against women is viewed as normative behavior [28].

Studies in Ethiopia have also shown that about one-half to two-third of women experience one or other forms of spousal abuse at least once in their lifetime [29–33]. Intimate partner violence affects the utilization of different reproductive health services in different countries [18–21, 34–41] including Ethiopia [31–33, 42–47].

In Ethiopia, different researches have been done on the prevalence and/or factors associated with women's attitude towards wife-beating. Age, residence, educational status, and religion are determinant factors identified by scholars [29–33, 42–46]. But, all the studies were done at a local level, use a small sample size, do not consider the effect of community-level factors on women's attitude towards wife-beating. Besides, the association at the individual-level may not work at the community level and vice versa. Even the studies were fitted with standard logistic regression which may lead to loss of power. “National representative evidence is important to achieve the national, international goals towards gender equity, equality, women empowerment and to ensure health for all”. Therefore, this study aimed to assess individual and community-level factors associated with acceptance of wife-beating among—women in Ethiopia by using EDHS 2016 will be important to develop community-level behavioral change communication to reduce the prevalence and impact of intimate partner violence in the country.

## Methods

### Study setting and period

The study was conducted in Ethiopia, which is located in the North-eastern (horn of) Africa, lies between 3^0^ and 15^0^ North latitude and 33^0^ 48^0^ and East longitudes. This study used 2016 EDHS data set which was collected by the Central Statistical Agency (CSA) [48]. Data were accessed from their URL: www.dhsprogram.com by contacting them through personal accounts after justifying the reason for requesting the data.

A total of 15,683 weighted reproductive age women were included in the analysis. EDHS 2016 sample was stratified and selected in two stages. In the first stage, stratification was conducted by region, and then each region stratified as urban and rural, yielding 21 sampling strata. A total of 645 (202 urban and 443 rural) enumeration areas [49] were selected with probability proportional to EA size in each sampling stratum. In the second stage affixed number of 28 households per cluster were selected with equal probability systematic selection from the newly created household listing.

### Variable measurement and definition

In this study the outcome variable (acceptance of women towards wife-beating) was dichotomized as (Yes/No). Reproductive age women acceptance towards wife-beating was measured by computing the following variables (burning food, arguing with husband, going out without telling husband, neglecting the children, and refusing to have sexual intercourse with her husband). If a women say “yes” at least one from the above five variables, she was considered as accepting wife-beating [48]. The independent variables were individual-level factors including (age, religion, wealth index, educational status, media exposure) and community-level factors were created by aggregating individual-level factors in each cluster (region, residence, community-level of education, community-level wealth index, community-level television exposure, and community-level radio exposure). The community-level of wealth index was generated by using the proportion of the two (poorest and poorer) lowest level of wealth index to the total wealth index of the same cluster. Similarly community-level of education is generated by using the proportion of the two (secondary and higher education) high level of educational attainment to the total the educational level of the same cluster. Community-level of television exposure is also computed by dividing exposed to television for the total respondents, Community-level radio exposure is computed by dividing exposed to radio to the total respondents. Since all the above four variables are not normally distributed, we were using the median as cutoff point (above median: women who live in a cluster with a high proportion of poor community, low community educational status, low community media exposure) to dichotomize the variables.

### Data processing and analysis

Data cleaning was conducted to check for consistency with the EDHS-2016 descriptive reports. Recoding, variable generation, labeling, and analysis were done by using Stata version 14.0. Descriptive statistics were done to describe the study participants in relation to socio-demographic characteristics which were presented in tables and text. Sample weight was used to compensate for the unequal probability of selection between the strata that were geographically defined and for non-responses. Multilevel analysis was conducted after checking the eligibility of the data to multilevel analysis by using the intra-cluster correction coefficient (ICC). When the ICC is greater than 10% (ICC = 23%), the community-level factors affect the dependent variable. Therefore it is better to identify community-level factors to develop and take different interventions. Since EDHS data are hierarchical (individual “level 1” were nested within a community “level 2”), a two-level mixed-effects logistic regression model was fitted to estimate both independent (fixed) effects of the explanatory variables and community–level random effects on acceptance of wife-beating among reproductive age women. The log of the probability of accepting wife-beating was modeled using a two-level multilevel model as follows: Log $\left[\frac{{\Pi_{ij} }}{{1 - \Pi_{ij} }}\right]$ = β_0_ + β_1_X_ij_ + B_2_ Z_ij_ + µ_j_ + e_ij_, Where i and j are individual level and community level [2] unites respectively; X and Z refers to individual and community level variables respectively; *πij* is the probability of accepting wife-beating for the ith women in the jth community; β’s indicates the fixed coefficients. (Β_0_) is the intercept, the effect on the probability of accepting wife-beating in the absence of influencing factors; and µj showed the random effect (the effect of the community on the acceptance of wife-beating of the jth community) and eij showed random errors at individual level. By assuming each community had a different intercept (Β_0_) and fixed coefficient (β), the clustered data nature and intra and inter-community variations were taken into account.

During analysis first, bi-variable multilevel logistic regression was fitted and variables with *p* value less than 0.2 at model I and model II were selected to develop the 3^rd^ model (the final model). The analysis was done in four models. The first model was, model-0 (empty model or null model/ without explanatory variable; to secure the need to multilevel analysis). The second model was, model-I (analyzing only individual-level variable), the 3rd model was, model-II (analyzing only community-level variable), the last model, model-III (analyzing both community level and individual level variables based on the cutoff point).

The measure of association (fixed effects) estimate the association between the likelihood of acceptance of wife-beating among reproductive age group women and different explanatory factors were expressed by Adjusted Odds Ratio (AOR) with respective 95% confidence level. Variables with *p* value less than 0.05 at model-III were significantly associated with acceptance of wife-beating. The random-effects (variations) were measured by using ICC (model-0), Median Odds Ratio (MOR) in (model-I and II), and Proportional Change in Variance (PCV) was measured to show variation between clusters.

ICC shows the variation in acceptance of wife-beating among women due to community characteristics. The higher the ICC, the community characteristics are more relevant to understand individual variation for acceptance of wife-beating. It is calculated as ${\text{ICC}} = \left( {\frac{{\delta^{2} }}{{\delta^{2} + \frac{{\pi^{2} }}{3}}}} \right)$, where δ^2^ indicates estimated variance of clusters. MOR is the median value of the odds ratio between the area at highest risk and the area the lowest risk when randomly picking out two areas and it was calculated as MOR = exp. ($\sqrt {2 \times \delta^{2} + .6745}$) ≈ exp^(0.95δ)^. In this study, MOR shows the extent to which the individual probability of accepting wife-beating for women determined by place of residence. PCV measures the total variation attributed by individual-level variables and area-level variables in the final model (model-III). It is calculated as PCV = [*δ*^2^ *of null model* − *δ*^2^ *of each model*)/*δ*^2^ *of null model*]. δ^2^ of the null model is used as a reference.

Multicollinearity was checked among explanatory variables by using standard error at a cutoff point ± 2. No Multicollinearity is the standard errors were between ± 2. The log-likelihood test was used to estimate the goodness of fit of the adjusted final model (model-III) in comparison to the preceding models (model-I and model-II) individual and community model adjustments respectively. The analysis was done by using the SVY Stata commands to control the clustering effect of complex sampling (stratification and multistage sampling procedures).

## Results

### Characteristics of the respondents

A total of 15,683 reproductive age [15–49] women were included in the analysis. Among this, 3380.9 (21.6%) were found in the age group of 15–19 years, 7497.9 (47.8%) study participants have not attended school. About 12,207 (77.8%) of women residing in rural areas. 11,359.5 (72.4%) of women live in areas of the low proportion educated community (Table 1).

**Table 1:** Individual and community-level characteristics of reproductive age women in Ethiopia, EDHS 2016 (n = 15,683)

Variable	Number	Percent
*Age*		
15–19	3380.9	21.6
20–24	2761.8	17.6
25–29	2956.7	18.9
30–34	2345.2	14.9
35–39	1932.1	12.3
40–44	1289.6	8.2
45–49	1016.7	6.5
*Religion*		
Orthodox	6786.2	43.3
Protestant	3674.1	23.4
Muslim	4892.7	31.2
Others*	330.0	2.1
*Educational status*		
No education	7497.9	47.8
Primary	5490.4	35.0
Secondary	1817.5	11.6
Higher	877.2	5.6
*Household wealth index*		
Poorest	2632.8	16.8
Poorer	2809.2	17.9
Middle	2978.2	19.0
Richer	3099.6	19.8
Richest	4163.2	26.7
*Marital status*		
Never married	4036.4	25.7
Married	10,453.7	66.7
Widowed/divorced	1192.9	7.6
*Number of living children*		
None	5185.3	33.1
1–2	3769.5	24.0
3–4	3063.7	19.5
5 and above	3664.5	23.4
*Frequency of watching television*		
Not at all	11,294.6	72.0
Less than once a week	1903.5	12.1
At least once a week	2484.9	15.9
*Frequency of listening radio*		
Not at all	10,485.4	66.9
Less than once a week	2616.7	16.7
At least once a week	2580.9	16.5
*Ever chewing chat*		
No	13,779.4	87.9
Yes	1903.6	12.1
*Residence*		
Urban	3476	22.2
Rural	12,207	77.8
*Region*		
Tigray	1129.0	7.2
Afar	128.2	0.8
Amhara	3714.1	23.7
Oromia	5700.9	36.4
Somali	459.5	2.9
Benishangul	160.4	1.0
SNNP	3288.0	21.0
Gambella	43.7	0.3
Harari	38.5	0.2
Addis Ababa	930.3	6.0
Dire Dawa	90.4	0.6
*Community-level of wealth*		
A low proportion of poor	9078.2	57.9
A high proportion of poor	6604.8	42.1
*Community-level of education*		
A high proportion of educated	4323.5	27.6
A low proportion of educated	11,359.5	72.4
*Community-level of television exposure*		
A low proportion of television exposure	3546.9	22.6
A high proportion of television exposure	12,136.1	77.4
*Community-level of radio exposure*		
A low proportion of radio exposure	5688	36.3
A high proportion of radio exposure	9995	63.7

### Individual and community level factors associated with women acceptance of wife beating

In the final model (model-III) educational status, religion, marital status, region, community-level wealth, and community-level of television exposure had a statistical association with women acceptance of wife beating.

The odds of women acceptance on wife beating was 1.3 times more among participants who are Muslim religion followers as compared to Orthodox religion followers [AOR = 1.3, 95% CI = (1.1, 1.5)]. Women who were attending primary education, secondary education and higher education were 20%, 60% and 70% less likely accept wife beating when compared with not attend school [AOR = 0.8, 95% CI = (0.7, 0.9)], [AOR = 0.4, 95% CI = (0.3, 0.5)], [AOR = 0.3, 95% CI (0.2, 0.4)] respectively.

The odds of women acceptance on wife beating was 1.3 times more among married women when compared with never married women [AOR = 1.3, 95% CI = (1.1, 1.6)]. Women who were living in Somali, Addis Ababa and Dire Dawa were 80%, 70% and 50% less likely accept wife beating when compare to women who live in Tigray region [AOR = 0.2 95% CI = (0.1, 0.3)], [AOR = 0.3, 95%CI = (0.2, 0.5)] and [AOR = 0.5, 95% CI = (0.3, 0.7)] respectively.

Women who live in a high proportion of poor communities were 1.2 times more likely accepted wife-beating than women who live in a low proportion of poor communities [AOR = 1.2, 95% CI = (1.1, 1.3)]. Women who live in a low proportion of television exposure communities were 1.4 times more accept wife-beating than women who live in a high proportion of television exposure community [AOR = 1.4, 95% CI = (1.2, 2.2)] (Table 2).

**Table 2:** Multilevel logistic regression analysis of individual and community-level factors associated with wife-beating acceptance among reproductive age women in Ethiopia, EDHS 2016 (n = 15,683)

Variable	COR (95%CI)	Model-0 ICC = 22.59%	Model-I (AOR) (95%CI)	Model-II (AOR) (95%CI)	Model-III (AOR) (95%CI)
*Age in years*					
15–19	1				
20–24	1.0 (0.8, 1.2)		1.0 (0.8, 1.2)		1.0 (0.8, 1.2)
25–29	1.1 (.9, 1.4)		0.9 (0.7, 1.2)		1.0 (0.8, 1.2)
30–34	1.3 (1.1, 1.5)		1.0 (0.7, 1.2)		1.0 (0.8, 1.3)
35–39	1.2 (1.0 1.5)		0.9 (0.7, 1.2)		1.0 (0.7, 1.3)
40–44	1.3 (1.1, 1.6)		1.0 (0.7, 1.3)		1.0 (0.8, 1.4)
45–49	1.1 (0.9, 1.5)		0.8 (0.6, 1.1)		0.9 (0.6, 1.2)
*Religion*					
Orthodox	1				
Protestant	1.2 (0.9, 1.5)		1.1 (0.9, 1.4)		1.1 (0.9, 1.4)
Muslim	1.5 (1.2, 1.8)		1.0 (0.9, 1.2)		**1.3 (1.1, 1.5)**
Others*	1.2(0.6, 2.2)		1.1 (0.5, 2.2)		1.1 (0.6, 2.2)
*Educational status*					
No education	1				
Primary	0.7 (0.6, 0.85)		0.8 (0.7, 0.9)		**0.8 (0.7, 0.9)**
Secondary	0.3 (0.2, 0.4		0.4 (0.3, 0.5)		**0.4 (0.3, 0.5)**
Higher	0.2 (0.1, 0.26)		0.3 (0.2, 0.4)		**0.3 (0.2, 0.4)**
*House hold wealth index*					
Poorest	1				
Poorer	1.2 (0.9, 1.4)		1.2 (1.0, 1.5)		1.2 (0.9, 1.4)
Middle	0.9 (0.7, 1.0)		1.0 (0.8, 1.2)		0.9 (0.7, 1.1)
Richer	0.7 (0.5, 0.8)		0.8 (0.7, 1.0)		0.8 (0.6, 1.0)
Richest	0.4 (0.3, 0.5)		0.7 (0.5, 0.9)		0.8 (0.6, 1.1)
Marital status					
Never married	1				
Married	1.5 (1.3, 1.7)		1.4 (1.1, 1.7)		**1.3 (1.1, 1.6)**
Widowed/divorced	1.5 (1.2, 1.9)		1.3 (0.9, 1.7)		1.2 (0.9, 1.6)
Number of living children					
None	1				
1–2	1.3 (1.1, 1.5)		0.9 (0.7, 1.1		0.9 (0.7, 1.1)
3–4	1.4 (1.2, 1.6)		0.8 (0.6, 1.1)		0.8 (0.6, 1.1)
5 and above	1.4 (1.2, 1.6)		0.8 (0.6, 1.0)		0.8 (0.6, 1.0)
*Frequency of watching television*					
Not at all	1				
Less than once a week	0.8 (0.6, 0.9)		1.0 (0.8, 1.2)		1.0 (0.8, 1.2)
At least once a week	0.4 (0.3, 0.5)		0.7 (0.6, 0.9)		0.8 (0.7, 1.1)
Frequency of listening radio					
Not at all 1					
Less than once a week	0.8 (0.6, 0.9)		1.0 (0.8, 1.1)		1.0 (0.8, 1.1)
At least once a week	0.6 (0.5, 0.7)		0.9 (0.7, 1.0)		0.9 (0.7, 1.0)
*Residence*					
Urban	1				
Rural	4.5 (3.7, 5.3)			1.4 (0.9, 2.2)	1.3 (0.8, 2.0)
*Region*					
Tigray	1				
Afar	1.2 (0.8, 1.8)			1.2 (0.9, 1.6)	0.8 (0.6, 1.1)
Amhara	1.1 (0.8,1.4)			0.9 (0.7, 1.2)	0.8 (0.6, 1.1)
Oromia	1.2 (0.9, 1.7			1.1 (0.9, 1.5)	0.9 (0.7, 1.2)
Somali	0.4 (0.3, 0.5)			0.3 (0.2, 0.4)	**0.2 (0.1, 0.3)**
Benishangul	0.6 (0.4, 0.9)			0.5 (0.4, 0.8)	**0.5 (0.3, 0.7)**
SNNP	0.9 (0.7, 1.3)			0.9 (0.7, 1.1)	0.8 (0.6, 1.0)
Gambela	0.79 (0.6, 1.1)			1.0 (0.7, 1.3)	0.9 (0.6, 1.3)
Harari	0.3 (0.2, 0.5)			0.5 (0.3,0.7)	**0.3 (0.2, 0.5)**
Addis Ababa	0.13 (0.1, 0.2)			0.4 (0.3 0.5)	**0.3 (0.2, 0.5)**
Dire Dawa	0.4 (0.3, 0.6)			0.7 (0.5, 1.0)	**0.5 (0.3, 0.7)**
*Community-level of wealth*					
A low proportion of poor	1				
A high proportion of poor	2.3(1.9, 2.8)			1.3 (1.1, 1.6)	**1.2 (1.1, 1.3)**
*Community-level of education*					
A high proportion of educated	1				
A low of educated	3.4 (2.8, 4.13)			1.1 (0.9, 1.4)	0.8 (0.7, 1.1)
*Community-level of television exposure*					
A high proportion of exposed	1				
A low proportion of exposed	4.4 (3.6, 5.2)			1.6 (1.1, 2.5)	**1.4 (1.2, 2.2)**
*Community-level of radio exposure*					
A high proportion of exposed	1				
A low proportion of exposed	2.4 (2.0, 2.9)			1.2 (0.98 1.5)	1.1 (0.9, 1.4)

^*^ = traditional, no religion and catholic, 1 = reference. Bold = significant factors at the final model

### Random effects (measures of variation)

Women's acceptance towards wife-beating varies significantly across each cluster. ICC indicated, 23.3% of the variation in acceptance of wife-beating among women was attributed to community-level factors. PCV in the final model shows 60% of the variation in acceptance towards wife-beating across communities was explained. Likewise, MOR for acceptance towards wife-beating among women, in the null model was 8.1 which shows the presence of variation across each cluster (Table 3).

**Table 3:** Measure of variation for acceptance of wife beating among reproductive age group women at cluster-level in multilevel logistic regression analysis, EDHS 2016

Measure of variation	Model-0 (null)	Model-I	Model-II	Model-III
Variance	1.0	0.6	0.5	0.4
ICC (%)	23.3	15.2	12.0	11.8
PCV (%)	Reference	40	50	60
MOR	8.1	6.4	3.7	2.5
*Model fitness*				
Log-likelihood	− 9283.8109	− 8981.6657	− 9116.8628	− 8900.3833

## Discussion

The result of the final model showed that individual-level factors:( educational status, religion, marital status) and community-level factors:( region, community-level of wealth, and community-level of television exposure) were determinant factors of wife-beating acceptance among reproductive age women in Ethiopia.

As educational attainment increases the acceptance of wife-beating is reduced. The finding is consistent with previous researches. In Ethiopia, refusing wife-beating was differed by educational attainment [32]. A study conducted in seven Asian countries also revealed that better-educated women were more likely to disapprove of wife-beating than less educated ones [50]. Moreover, researches conducted in Israel [51], Korea [52], India [53], Palestine [13], and seventeen sub-Saharan African countries (including Ethiopia) [54] had also come up with consistent findings. According to WHO, education is the strongest demographic predictor explaining wife-beating attitudes [55]. This might be due to Education, being a mechanism of acquiring knowledge, developing common understanding, and enhancing decision-making autonomy, it appeared to have a direct relationship with resistances against wife-beating [32]. Research findings reveal that better-educated women have not accepted the socio-cultural settings of the society rather guided by logic and common understanding based on modern and rational thinking [13, 32, 56, 57]. The inverse relationship between educational attainment and dependency syndrome of wives on their husbands to earn their living has also accelerated a better-educated women’s resistance to wife-beating [56, 58, 59]. It is clearly identified that education is not only one of the best indicators of women’s status in society, but also the other effective and powerful means of ending violence against women [60]. The direct relationship between education and resistance against wife-beating is also a reflection of education’s power to diminish socio-cultural factors [61] that perpetuate males in traditional societies like Ethiopia. Moreover, Educated women are likely to be in a more modern and egalitarian relationship than uneducated women with their husbands [62]. Currently, girls’ education is on the government priority agenda in Ethiopia. Therefore, the proportion of educated young women is expected to rise and to continue having an impact on the reduction of acceptance of wife-beating in the future.

Muslim religion follower women were more accepting wife-beating than Orthodox religion follower women. Similarly, in previous research conducted in Ethiopia, Muslims are accepting wife-beating than Orthodox [32]. In research among seven sub-Saharan African countries, attitude towards wife-beating and religion, Muslims in Mali and Benin were more likely to justify wife-beating [54]. A study in Ghana also revealed that compared to Christian women, Muslims were more likely to approve physical violence against wives [63]. This might be, religion tends to clutch abundant moral values affecting the power relation between husband and wife [14, 64–66]. More religious women are believed to hold the conservative and traditional belief that stimulates husbands’ to abuse their wives [67]. Potential explanations include the influence of religious traditions, teachings, doctrines, religious communities, and institutions that convey values and belief systems to their members.

Currently married women tend to accept wife-beating than never-married women. The finding is supported by research in Ethiopia [32]. This is believed to be the effect of the socio-cultural influences which have been supported by traditional norms and values that have allowed Ethiopian males to ‘discipline’ their wives and correct them to their wants. It may be that ever-married women, having experienced domestic violence firsthand, might respond to the EDHS that fits in with community attitudes. By contrast, never-married women may have better knowledge about rights, gender equality, and female empowerment, which could encourage this group of women to challenge traditional norms that require women to be submissive to their husbands. Never-married women are more likely to be educated than their never-married counterparts. This suggests that these attitudes are modifiable and that community norms and public discourse are powerful determinants of individual attitudes and values about gender-based norms [38, 68].

There is a regional variation in acceptance of wife-beating among women. Women who live in Addis Ababa, Dire Dawa, Harari, and Somali region are less likely to accept wife-beating when compared with women who live in Tigray region. Cultural, religious values, and norms may be different across the regions [69]. Cultural norms, social changes, family dynamics, and government policies influence attitude [13, 32, 38, 57, 70]. Living in urban areas facilitates exposure to people from diverse backgrounds, living arrangements, and lifestyles which, in turn, can promote gender-egalitarian views [49, 71].

When a high proportion of poor people lived in the cluster, the acceptance of wife-beating increased. This is also supported by a study conducted in sub-Saharan African countries [62, 72]. This might be due to rich communities can offer greater opportunities for higher education and employment [73–76]. This, in turn, can positively impact women’s attitudes [70]. Research documents, lower levels of wife-beating acceptance among those living in advantaged communities [38, 57, 68]. Moreover, women in the poor community might face economic, social, and educational constricts to challenge the norms which force them to accept existing social views [49, 77–80].

When a low proportion of people espoused to television in the cluster, the acceptance of wife-beating increased. The finding is consistent with a search conducted in Ethiopia [42] and seventeen countries of sub Saharan Africa [54]. That revealed that women who live in a cluster of low access to media information were more supportive of wife-beating than their counterparts. This might be, Media exposure creates awareness about the existing law against women discrimination in the country which is a key factor influencing the tendency of women to believe wife-beating is justified [17, 81, 82]. More specifically, the study confirms that women who lack awareness of the existing law that prohibits wife-beating in Ethiopia are most likely to support wife-beating than women who are aware of the existing law [42]. Media espouse might change the community and women's believes that women benefit and gain from violence [83].

The result of this study was more representative than other studies and the model considered different levels of analysis as the outcome was affected by community-level variables. Despite this strength, the result may be prone to recall bias because the data were collected from history of event.

## Conclusion

After computing multi-level analysis, low educational status, being married, Muslim religion followers, region, live in high a proportion of poor community, live in a high proportion of non-television exposed community had a statistical association with acceptance of wife-beating among reproductive age group women in Ethiopia. Improving universal access to education is important to reduce the prevalence as well as health and health-related complications of the acceptance of wife-beating. Advocacy and behavioral change communication should be area of concern for different organizations who are working on women reproductive health to tackle the acceptance as well as the consequences of gender-based violence. Since the acceptance of wife-beating is different across community, better to develop community sensitive approaches for different communities.

## Data Availability

The datasets used and/or analysed during this study are available from the corresponding author on reasonable request.

## Abbreviations

CSA: Central statistics agency
EA: Enumeration area
ICC: Inter cluster coefficient
MOR: Median odds ratio
PCV: Proportional change variance
